# Neutrophils Activate Tumoral CORTACTIN to Enhance Progression of Orohypopharynx Carcinoma

**DOI:** 10.3389/fimmu.2013.00033

**Published:** 2013-02-18

**Authors:** Claudia A. Dumitru, Agnes Bankfalvi, Xiang Gu, Wilfried E. Eberhardt, Reinhard Zeidler, Stephan Lang, Sven Brandau

**Affiliations:** ^1^Department of Otorhinolaryngology, University Hospital Essen, University of Duisburg-EssenEssen, Germany; ^2^Department of Pathology/Neuropathology, University Hospital Essen, University of Duisburg-EssenEssen, Germany; ^3^Department of Medical Oncology, West German Cancer Centre, University Hospital Essen, University of Duisburg-EssenEssen, Germany; ^4^Department of Otorhinolaryngology, Ludwig Maximilians UniversityMunich, Germany

**Keywords:** cancer-related inflammation, tumor microenvironment, head and neck cancer, clinical outcome, tumor migration

## Abstract

CORTACTIN is an actin-binding protein critically involved in cellular migration and invasion. Here, we investigated the role of CORTACTIN in the pathophysiology of orohypopharynx carcinoma – one of the major subtypes of head and neck cancer. To this end, we analyzed CORTACTIN expression in tumor tissues from 89 orohypopharynx carcinoma patients in relation to clinical parameters. We found that high tumoral CORTACTIN expression associated with poor survival, higher T-stage, and higher lymph node metastasis (N-stage) in these patients. Next, we combined the prognostic values of tumoral and stromal cell biological parameters in our patient cohort. We determined the potential interaction of tumoral CORTACTIN with tumor-infiltrating neutrophils, which have been previously linked to poor clinical outcome in orohypopharynx carcinoma patients with advanced disease. Interestingly, we found that patients with both high tumoral CORTACTIN expression and high neutrophilic infiltration had significantly worse clinical outcome than all other patients in our cohort. These findings suggest that tumoral CORTACTIN and tumor-infiltrating neutrophils might be functionally linked during progression of orohypopharynx carcinoma. *In vitro*, we showed that neutrophils released soluble factors which phosphorylated CORTACTIN in the tumor cells and promoted their migration. Furthermore, we demonstrated that strong CORTACTIN phosphorylation significantly correlated with strong neutrophilic infiltration in tumor tissues from orohypopharynx carcinoma patients. Taken together, our findings unravel a novel mechanism of tumor-stroma interaction, which might be relevant for a more accurate prognosis and improved therapeutic strategies in this tumor entity.

## Introduction

Head and neck cancer (HNC) is the eighth most common type of cancer worldwide. Despite multiple and aggressive therapeutic interventions, there has been no fundamental improvement in the 5-year survival rates of the patients over the past decades (Jemal et al., 2007; Choong and Vokes, 2008). Therefore, there is an urgent need to: (i) identify clinicopathological parameters (biomarkers) for accurate diagnosis, prognosis, and therapeutic prediction in these patients and to (ii) understand the biology and molecular mechanisms behind the respective biomarkers.

A number of studies investigated the role of cortical actin-binding protein (CORTACTIN) as prognostic marker in different types of cancer. CORTACTIN is a cytoplasmic protein which promotes the rearrangement of the actin cytoskeleton. Consequently, CORTACTIN is critically involved in regulation of cellular motility/migration, invasion, or endocytosis/vesicular trafficking (reviewed in Ammer and Weed, 2008). To exert its functions, CORTACTIN needs to be activated by tyrosine or serine/threonine phosphorylation. In particular, tyrosine phosphorylation of CORTACTIN at residues Tyr 421, Tyr 466, and Tyr 482 has often been shown to occur in response to various stimuli (Ammer and Weed, 2008). Consistent with its role in cellular migration and invasion, high CORTACTIN expression and/or activation has been found to associate with increased metastasis and poor clinical outcome in several types of cancer, such as renal carcinoma (Wang et al., 2009), gastric cancer (Li et al., 2008), colorectal adenocarcinoma (Cai et al., 2010), or melanoma (Xu et al., 2010). In HNC, amplification of CORTACTIN gene has been linked to poor clinical outcome mainly in patients with laryngeal carcinoma (Gibcus et al., 2008; Rodrigo et al., 2009).

Solid tumors display an inflammatory microenvironment, characterized by large numbers of tumor-associated immune cells, which represent a biologically important component of the tumor-stroma (Coussens and Werb, 2002; Lin and Karin, 2007). Within the tumor microenvironment, cancer cells can “reprogram” the stromal immune cells of the host to acquire tumor-promoting activities. Although less characterized than tumor-associated macrophages (TAMs) or tumor-infiltrating lymphocytes (TILs), tumor-infiltrating neutrophils are emerging as important players in the pathophysiology of cancer. Within the tumor tissue, neutrophils can potentially modulate multiple cellular processes which may ultimately lead to tumor progression. In particular, neutrophils were shown to promote angiogenesis (Nozawa et al., 2006; Jablonska et al., 2010; Bekes et al., 2011; Kuang et al., 2011). However, neutrophils can directly modulate the biology and functions of tumor cells by promoting their migration, invasion, or proliferation (reviewed in Gregory and Houghton, 2011; Dumitru et al., 2012b). Thus, it is not surprising that very recent studies reported an association of high numbers of tumor-infiltrating neutrophils with advanced disease and poor clinical outcome in patients with different types of cancer, such as renal cancer (Donskov and von der Maase, 2006; Jensen et al., 2009), hepatocellular cancer (Kuang et al., 2011), non-small-cell lung carcinoma (NSCLC; Ilie et al., 2012), or melanoma (Jensen et al., 2012). Recently, we demonstrated that high neutrophilic infiltration of the tumor tissue associated with high tumor (T) stage and poor survival in head and neck (orohypopharynx) cancer patients with advanced disease (Trellakis et al., 2011). Furthermore, our *in vitro* studies indicated a direct interaction between neutrophils and HNC cells by showing that neutrophils were “primed” by the tumor cells to release pro-inflammatory factors which promoted tumoral migration in a feed-back manner (Dumitru et al., 2011, 2012a).

In the present study we characterized the tumor-neutrophil interactions in orohypopharyngeal cancer with particular focus on the role of tumoral CORTACTIN in this process. We demonstrate that high tumoral CORTACTIN levels together with high neutrophilic infiltration predict poor clinical outcome in univariate and multivariate analysis. These findings suggest that tumoral CORTACTIN and tumor-infiltrating neutrophils “cooperate” in the progression of orohypopharynx carcinoma. Further *in vitro* and *in situ* studies confirm the clinical findings and demonstrate that neutrophils activate (phosphorylate) CORTACTIN in the tumor cells to promote their migration.

## Materials and Methods

### Study subjects

Neutrophils were isolated from the peripheral blood of healthy volunteers. For immunohistochemical studies on frozen sections, malignant tissues were collected from 37 patients with orohypopharyngeal cancer, treated at the Department of Otorhinolaryngology (University of Duisburg-Essen) between 2010 and 2012. Healthy epithelial tissues (*n* = 12) were collected from contralateral buccal mucosa of the same patients. For immunohistochemical studies on tissue microarrays (TMAs) and correlation with clinical parameters, tissue samples were collected from 89 patients with head and neck squamous cell carcinoma of the oropharynx and hypopharynx. The patients were treated at the Department of Otorhinolaryngology (University of Duisburg-Essen) between 1995 and 2000 and clinical follow-up was retrieved. The characteristics of these patients are shown in Table 1. All studies were approved by the ethics committee of the University Hospital Essen (nr. 09-4074, nr. 07-3500, and nr. 12-5192-BO).

**Table 1:** **Characteristics of the patients used for TMA analysis and association with clinical parameters (survival, T-stage, and N-stage)**.

	Number	% of total
All patients	89	100
**GENDER**		
Male	71	79.8
Female	18	20.2
**TUMOR LOCALIZATION**		
Oropharynx	65	73.0
Hypopharynx	24	27.0
**T-STAGE**		
T1	13	14.6
T2	19	21.3
T3	12	13.5
T4	45	50.6
**N-STAGE**		
NO	14	15.7
N1	13	14.6
N2a	6	6.7
N2b	20	22.5
N2c	32	36.0
N3	4	4.5
**DISTANT METASTASIS**		
MO	86	96.6
M1	3	3.4
**HISTOLOGIC GRADE**		
Grade 1	6	6.7
Grade 2	55	61.8
Grade 3	28	31.5

### Cell lines, conditioned supernatants, and siRNA transfections

The human hypopharyngeal carcinoma cell line FaDu was obtained from the American Type Culture Collection (ATCC, Manassas, VA, USA). The cells were cultured and maintained in RPMI-1640 (Invitrogen, Karlsruhe, Germany) supplemented with 10% fetal calf serum (Biochrom, Berlin, Germany), 100 units/mL penicillin, and 100 (g/mL streptomycin (PAA-Laboratories GmbH, Coelbe, Germany). To obtain tumor-conditioned supernatants (FaDu SN), we incubated 2 × 10^6^ cells/mL for 24 h at 37°C in RPMI-1640 supplemented as above. The resulting supernatant was centrifuged to remove cellular debris and stored at −20°C.

To knock-down CORTACTIN, we transfected FaDu cells with 25 nM validated CORTACTIN siRNA or 25 nM AllStars Negative Control (mock) siRNA (both from Qiagen, Hilden, Germany) using the NEON™ transfection system (Invitrogen, Karlsruhe, Germany).

### Migration assay

Migration of FaDu cells was assessed with the ORIS™ cell migration system (Platypus Technologies, Madison, WI, USA) according to the manufacturer’s instructions. The cells were allowed to migrate for 48 h and were then fixed/stained with a solution containing 2% formaldehyde, 30% ethanol, 60 mM NaCl, and 0.7% crystal violet. Micrographs were taken at 25× magnification and quantification of “gap”-closure was performed with the ImageJ software.

### Isolation and culture of neutrophils

Neutrophils (purity >98%) were isolated from the blood of healthy donors as previously described (Dumitru et al., 2011) and were cultured in RPMI-1640 supplemented as above.

### AMIDA screening

Tumors elicit an immune response, leading to the generation of antibodies specific for tumoral antigens. AMIDA technology (autoantibody-mediated identification of antigens) identifies proteins exclusively recognized by antibodies from cancer patients but not from healthy controls. Here, we used AMIDA to screen for tumor-associated antigens in four HNC patients versus four healthy donors. The screening was performed exactly as described previously (Rauch et al., 2004).

### Antibodies and inhibitors

Polyclonal rabbit anti-human CORTACTIN and rabbit IgG antibodies were obtained from Santa Cruz Biotechnology (Santa Cruz, CA, USA). Rabbit anti-human phospho-CORTACTIN (Tyr421) antibodies were from Cell Signaling Technology (Danvers, MA, USA) or from Millipore (Temecula, CA, USA). Rabbit anti-human GAPDH was from Cell Signaling Technology. Mouse anti-human CD66b antibodies were obtained from Immunotech (Marseille Cedex 9, France). The secondary antibodies (DyLight 488-donkey anti-rabbit, HRP-goat anti-rabbit, HRP-donkey anti-goat, AP-goat anti-rabbit) were from Dianova (Hamburg, Germany) while Alexa 546-goat anti-mouse IgG was from Molecular Probes (Leiden, The Netherlands). Protease inhibitor cocktail sets I and III were from Merck (Darmstadt, Germany). The phosphatase inhibitor cocktail PhosStop was from Roche Diagnostics (Mannheim, Germany).

### Immunohistochemistry and immunofluorescence

Frozen sections of tumor or healthy epithelial tissue were fixed with BD Cytofix/Cytoperm and incubated with primary antibodies overnight at 4°C. Secondary (and tertiary reactions, for colorimetric immunohistochemistry) were performed for 30 min each at room temperature. Nuclei were counterstained using Hematoxylin – for immunohistochemistry (Thermo Scientific, Karlsruhe, Germany) or DRAQ5 – for immunofluorescence (eBioscience, San Diego, CA, USA). Samples were finally mounted in Kaisers glycerol gelatine (Merck, Darmstadt, Germany) or in Fluoprep (bioMerieux, Marcy l’Etoile, France) and analyzed with a Zeiss Axioskop 2 microscope (Zeiss, Jena, Germany).

For TMAs, immunohistochemical staining was performed with an automated staining device (Dako Autostainer; DakoCytomation, Hamburg, Germany) using rabbit anti-human CORTACTIN antibodies. Secondary and tertiary immunoreactions were performed with commercially available anti-rabbit IgG detection kit (En-Vision; DakoCytomation, Hamburg, Germany). Analysis was performed with a Zeiss Axioscope 2 microscope. Staining and analysis of TMAs with CD66b antibodies has been described previously (Trellakis et al., 2011).

### Flow cytometry

To assess the levels of CORTACTIN after siRNA transfection, FaDu cells were fixed with BD Cytofix/Cytoperm and incubated with rabbit anti-CORTACTIN or isotype control antibodies for 1 h at room temperature. Secondary reactions were performed for 20 min at room temperature using DyLight488-conjugated anti-rabbit antibodies. Samples were analyzed by flow cytometry with a BD FACSCanto II flow cytometer (BD, Heidelberg, Germany).

### SDS-PAGE and Western blot analysis

FaDu cells (10^6^ cells/sample) were lysed with a buffer containing 25 mM HEPES (pH 7.3), 0.1% sodium dodecylsulfate (SDS), 1% Triton X-100, 10 mM EDTA, 10 mM sodiumpyrophosphate, 10 mM NaF, 125 mM NaCl, 1% protease inhibitor cocktail I, 1% protease inhibitor cocktail III, and 10% PhosStop. Cell debris was removed by centrifugation and the lysates were incubated with SDS-sample buffer (final concentrations 50 mM Tris pH 6.8, 4% glycerin, 0.8% SDS, 1.6% β-mercaptoethanol, and 0.04% bromophenol blue). Samples were further processed by SDS-PAGE and immunoblotting, respectively, as previously described (Dumitru et al., 2012a).

### Statistical analysis

For the *in vitro* studies, data are presented as means and SD and statistical analysis was performed with the two-tailed paired Student’s *t*-test. Clinical data was analyzed with the SPSS statistical software version 18.0 (SPSS Inc, Chicago, IL, USA). Survival curves (5-years cutoff) were plotted according to the Kaplan–Meier method and significance was tested using univariate analysis (log-rank test) or multivariate Cox regression analysis. Correlation between CORTACTIN phosphorylation and neutrophilic infiltration was tested with Spearmans’s rank correlation coefficient (Spearman ρ). Comparison of: (i) CORTACTIN expression with clinicopathological variables [T-stage and lymph node metastasis (N-stage)] or (ii) CORTACTIN phosphorylation with high versus low neutrophilic infiltration, respectively, was performed with the χ^2^ test. In all studies, the level of significance was set at *p* ≤ 0.05.

## Results

### CORTACTIN in orohypopharynx carcinoma: Expression and scoring system

**Figure 1:**
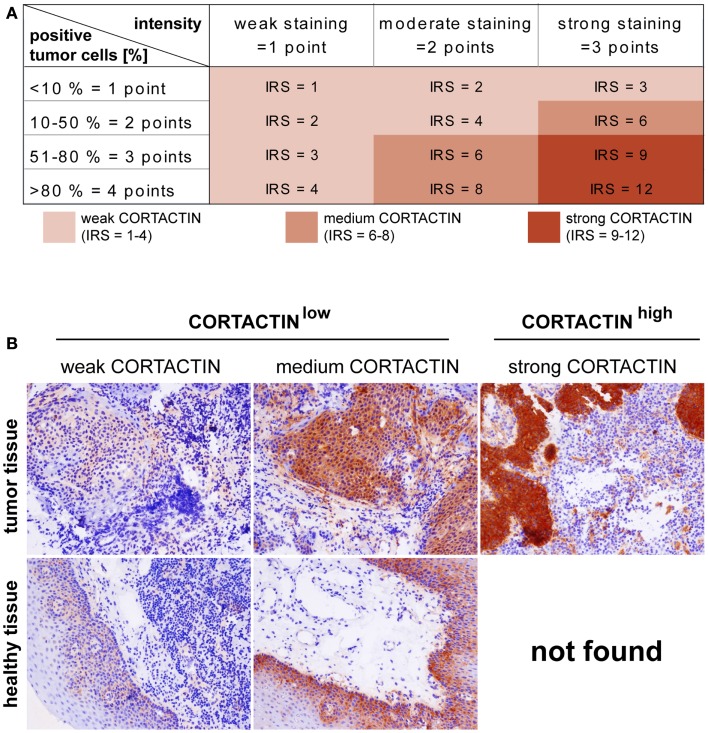
**CORTACTIN expression and scoring in tissue microarrays**. Malignant orohypopharyngeal and healthy epithelial tissues were stained against CORTACTIN. **(A)** The immunoreactive score (IRS) was calculated as intensity of the staining reaction multiplied by the percentage of positive cells. Based on the IRS values, CORTACTIN was scored as *weak*, *medium*, and *strong*. **(B)** Representative micrographs indicating that *strong* levels of CORTACTIN are found only in malignant tissues, while healthy epithelial tissues displayed either *weak* or *medium* levels of CORTACTIN. Subsequently, the weak and medium CORTACTIN-expressing specimens were considered as “CORTACTIN^low^” while strong CORTACTIN-expressing specimens were termed “CORTACTIN^high^.” Magnification = 200×.

Using AMIDA technology (Rauch et al., 2004) we initially screened for potential tumor-associated antigens in HNC patients (see Materials and Methods). Subsequently, we performed an extensive literature search to select the antigens which might enhance the aggressiveness and progression of tumors. Thus, we identified CORTACTIN as a promising candidate. We then determined the expression of CORTACTIN by immunohistochemical analysis of TMAs from 89 orohypopharynx carcinoma patients. Additionally, we determined CORTACTIN expression in frozen orohypopharynx carcinoma tissues (*n* = 16) versus healthy epithelial tissues from buccal mucosa (*n* = 12). CORTACTIN expression was scored as intensity of staining multiplied by the percentage of positive cells [immunoreactive score (IRS); Figure 1A]. An IRS of 1–4 was considered as *weak* CORTACTIN, IRS 6–8 as *medium*, and IRS 9–12 represented *strong* CORTACTIN levels (Figure 1A). Interestingly, in healthy epithelial tissues we observed *weak* and *medium* levels of CORTACTIN, but no *strong* CORTACTIN expression (Figure 1B). Consequently, we considered that *weak* and *medium* CORTACTIN represent basal levels of the protein (from here on termed “CORTACTIN^low^”), while *strong* CORTACTIN represents overexpressed levels of the protein (from here on termed “CORTACTIN^high^”).

### CORTACTIN and clinical outcome in orohypopharynx carcinoma patients

**Figure 2:**
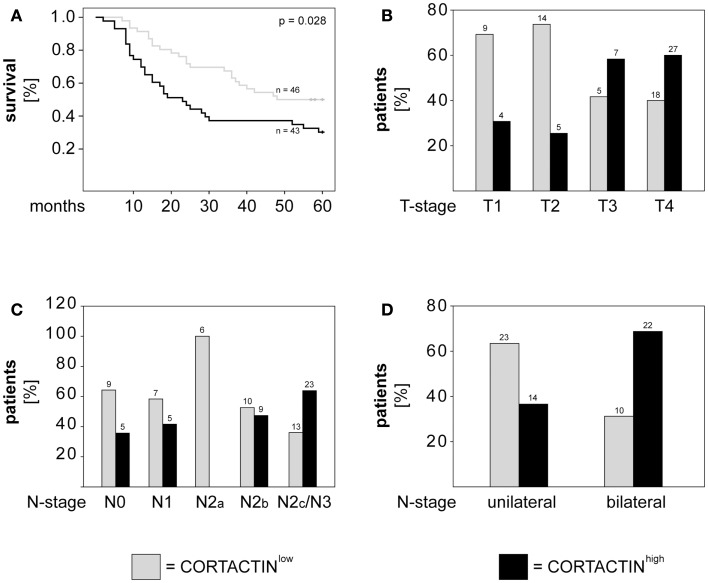
**CORTACTIN overexpression associates with advanced disease and poor clinical outcome in orohypopharynx carcinoma patients**. **(A)** Kaplan–Meier 5-years survival curves were plotted for patients with low versus high tumoral CORTACTIN expression and statistical analysis was performed with the log-rank test. Graphical depiction of **(B)** T-stage, **(C)** N-stage, and **(D)** laterality of lymph node metastasis in patients with low CORTACTIN versus high CORTACTIN expression. Statistical analysis was performed with the χ^2^ test (*p* = 0.027 for T2 versus T4; *p* = 0.008 for T1–2 versus T3–4; *p* = 0.005 for N2a versus N2c-3; *p* = 0.016 for N unilateral versus bilateral). The absolute patient numbers are indicated above each bar of the graphs.

Next, we determined whether CORTACTIN levels associated with 5-years survival, T-stage, and lymph node metastasis (N-stage) in orohypopharynx carcinoma patients. Here and throughout the whole study, T-stage was expressed as T1, T2, T3, T4, while N-stage was expressed as either N0, N1, N2a, N2b, N2c/N3 (advanced regional lymphadenopathy), or as unilateral (N1, N2a, N2b) versus bilateral (N2c) lymph node metastasis. The results demonstrated that patients with high levels of tumoral CORTACTIN had significantly shorter overall survival (*p* = 0.028, log-rank) than patients with CORTACTIN^low^ (Figure 2A). Furthermore, χ^2^ analysis indicated that patients with advanced disease were more likely to express high levels of CORTACTIN than patients with earlier stages of disease (*p* = 0.027 for T2 versus T4; *p* = 0.008 for T1–2 versus T3–4; *p* = 0.005 for N2a versus N2c-3; *p* = 0.016 for N unilateral versus bilateral). The distribution of patients with high and low expression of CORTACTIN in relation to T-stage and N-stage is depicted in Figures 2B–D.

### CORTACTIN and neutrophilic infiltration in orohypopharynx carcinoma

**Figure 3:**
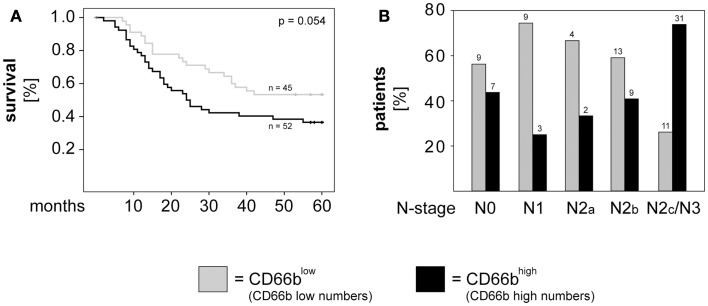
**High neutrophilic infiltration associates with poor survival and metastasis to the lymph nodes in orohypopharynx carcinoma patients**. **(A)** Kaplan–Meier 5-years survival curves were plotted for patients with low versus high neutrophilic infiltration and statistical analysis was performed with the log-rank test. **(B)** Graphical depiction of N-stage in patients with high versus low neutrophilic infiltration. Statistical testing was performed with the χ^2^ test (*p* = 0.005 for N1 versus N2c/N3; *p* = 0.015 for N2b versus N2c/N3; *p* = 0.001 for N unilateral versus bilateral). Patients with high numbers of infiltrating neutrophils (CD66b^high^) are depicted in black, while patients with low numbers of infiltrating neutrophils (CD66b^low^) are depicted in gray. The absolute patient numbers are indicated above each bar of the graphs.

Accumulating evidence indicates a critical role of tumor-infiltrating immune cells in the progression of different types of solid cancers. Recently, we demonstrated that high numbers of tumor-infiltrating neutrophils (CD66b^high^) associated with higher T-stage and poor survival in orohypopharynx carcinoma patients with advanced disease (Trellakis et al., 2011). Evaluation of the entire cohort of or hypopharynx carcinoma patients showed that high neutrophilic infiltration tended to associate with poor survival in those patients (*p* = 0.054; Figure 3A). Additionally, we found that patients with advanced N-stage were more likely to exhibit high neutrophilic infiltration than patients with earlier N-stages (*p* = 0.005 for N1 versus N2c/N3; *p* = 0.015 for N2b versus N2c/N3; *p* = 0.001 for N unilateral versus bilateral; χ^2^). The distribution of patients with high and low neutrophilic infiltration in relation to N-stage is depicted in Figure 3B.

**Figure 4:**
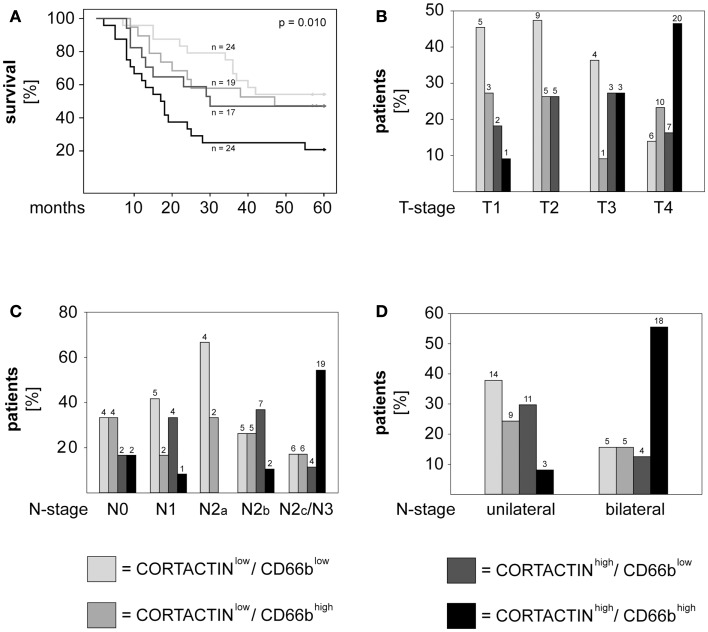
**High CORTACTIN and high neutrophilic infiltration (CD66b) associate with the most unfavorable clinical outcome**. Analysis of **(A)** 5-years survival, **(B)** T-stage, **(C)** N-stage, and **(D)** laterality of lymph node metastasis in patients with various combinations of CORTACTIN levels and neutrophilic infiltration, respectively. Statistical testing was performed with the log-rank test (for survival) and with the χ^2^ test for the remaining clinical parameters (*p* = 0.036 for T1 versus T4; *p* = 0.041 for T2 versus T3; *p* < 0.001 for T2 versus T4; *p* < 0.001 for T1–2 versus T3–4; *p* = 0.042 for N0 versus N2c/N3; *p* = 0.007 for N1 versus N2c/N3; *p* = 0.023 for N2a versus N2c/N3; *p* = 0.003 for N2b versus N2c/N3; *p* < 0.001 for N unilateral versus bilateral). The absolute patient numbers are indicated above each bar of the graphs.

Next, we investigated the combined effect of CORTACTIN and neutrophilic infiltration on the clinical outcome of orohypopharynx carcinoma patients. To this end, we divided the patients into four groups (CORTACTIN^low^/CD66b^low^, CORTACTIN^low^/CD66b^high^, CORTACTIN^high^/CD66b^low^, and CORTACTIN^high^/CD66b^high^) and determined the survival, T-stage, and the rate of lymph node metastasis. The results demonstrated that CORTACTIN^high^/CD66b^high^ patients had the shortest overall survival (*p* = 0.010, log-rank; Figure 4A). In further analysis we performed χ^2^ tests for CORTACTIN^high^/CD66b^high^ patients versus the other three groups of patients taken together. The results indicated that patients with advanced T-stage were more likely to express synchronous high levels of CORTACTIN and neutrophilic infiltration than patients with earlier stages of disease (*p* = 0.036 for T1 versus T4; p = 0.041 for T2 versus T3; *p* < 0.001 for T2 versus T4; *p* < 0.001 for T1–2 versus T3–4). Similar results were obtained for patients with advanced N-stage (*p* = 0.042 for N0 versus N2c/N3; *p* = 0.007 for N1 versus N2c/N3; *p* = 0.023 for N2a versus N2c/N3; *p* = 0.003 for N2b versus N2c/N3; *p* < 0.001 for N unilateral versus bilateral). The distribution of patients with various combinations of CORTACTIN/CD66b levels in relation to T-stage and N-stage is depicted in Figures 4B–D. To confirm the prognostic value of CORTACTIN and neutrophilic infiltration for the survival of orohypopharynx carcinoma patients, we performed multivariate Cox regression analysis in a model adjusted for the type of therapy received, smoking/alcohol consumption and gender. The results showed that the CORTACTIN^high^/CD66b^high^ phenotype was the only significant predictor for poor survival in these patients (HR = 2.34, 95% CI = 1.01–5.38, *p* = 0.045; Table 2). Taken together these data indicate that only the simultaneous presence of high tumoral CORTACTIN levels and high numbers of neutrophils associates with poor clinical outcome in orohypopharynx carcinoma patients. Furthermore, our findings suggest that CORTACTIN and neutrophils might functionally “cooperate” to enhance the progression of orohypopharynx carcinoma *in vivo*.

**Table 2:** **Multivariate Cox regression analysis of CORTACTIN and neutrophilic infiltration adjusted for type of therapy, smoking/alcohol consumption, and gender**.

Cox regression multivariate	Hazard ratio for survival		
	HR	95% CI	*p*-Value
**CORTACTIN/CD66b**			
CORTACTIN^low^/CD66b^low^	1		
CORTACTIN^low^/CD66b^high^	1.26	0.50–3.19	0.617
CORTACTIN^high^/CD66b^low^	1.37	0.54–3.50	0.502
CORTACTIN^high^/CD66b^high^	2.34	1.01–5.38	0.045*
**THERAPY**			
Surgery	1		
Radio(chemo)therapy	1.61	0.41–6.36	0.493
Surgery + radio(chemo)therapy	1.34	0.36–4.98	0.662
**ALCOHOL/TOBACCO**			
None	1		
Tobacco	0.97	0.28–3.37	0.970
Alcohol	0.61	0.06–6.12	0.611
Both	1.39	0.57–3.79	0.518
**GENDER**			
Female	1		
Male	2.04	0.88–4.73	0.093

HR, hazard ratio; 95% CI, 95% confidence interval. The asterisk (*) indicates the parameters showing statistical significance (*p* ≤ 0.05).

### Tumoral CORTACTIN and neutrophils: Mechanisms of interaction

**Figure 5:**
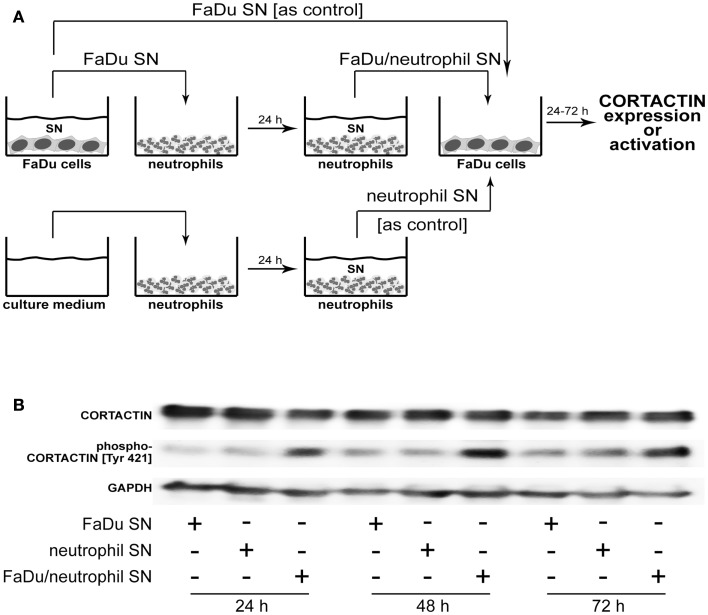
**Neutrophils phosphorylate CORTACTIN in FaDu (hypopharynx carcinoma) cells**. **(A)** Neutrophils were “primed” by co-incubation with FaDu supernatants (FaDu SN) for 24 h. The resulting supernatants (FaDu/neutrophil SN) were used to stimulate FaDu cells for 24, 48, or 72 h. FaDu SN or neutrophil SN only were used as controls. **(B)** CORTACTIN expression and activation were assessed by western blot using antibodies against total CORTACTIN (upper panel) or phosphorylated CORTACTIN (Tyr 421; middle panel). GAPDH (lower panel) was used as control for the quantity and quality of protein lysates. The results are representative for five independent experiments each.

In a further set of studies we sought to clarify the relationship between tumoral CORTACTIN and neutrophils by using an *in vitro* system well established in our group and previously described (Dumitru et al., 2011). This system mimics the reciprocal (bi-directional) interaction of tumor cells and neutrophils in the tumor microenvironment. As illustrated in Figure 5A, neutrophils are “primed” by tumoral factors and, subsequently, exert feed-back effects on the tumor cells (Figure 5A). We initially tested whether neutrophils might upregulate the expression of CORTACTIN in the tumor cells. To this end, FaDu cells (hypopharynx carcinoma) were stimulated with factors released by the “primed” neutrophils (FaDu/neutrophil SN). As control we used supernatants containing only tumoral factors (FaDu SN) or supernatants from “un-primed” neutrophils (neutrophil SN). We initially assessed the total levels of CORTACTIN in a time-course by western blot. We did not observe a significant change in CORTACTIN expression upon stimulation by neutrophils (Figure 5B – upper panel). However, when the samples were tested for phosphorylated CORTACTIN, we found increased levels of phospho-CORTACTIN in tumor cells exposed to factors released by the “primed” neutrophils (Figure 5B – middle panel).

**Figure 6:**
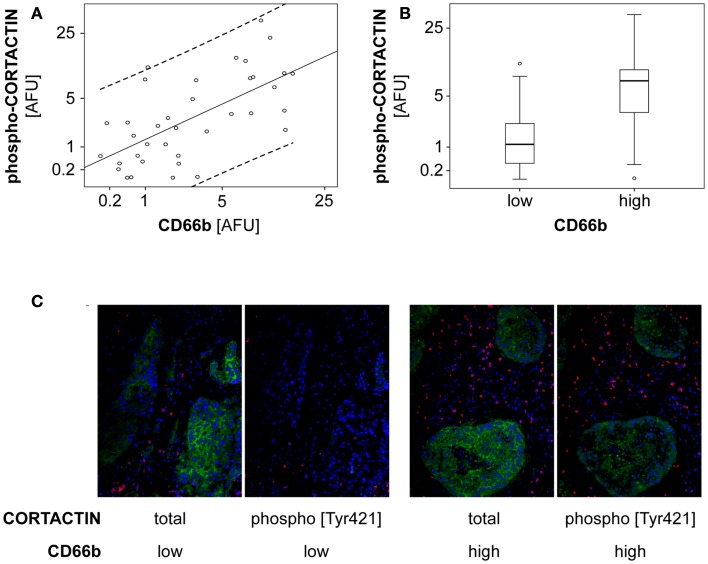
**Phosphorylated CORTACTIN associates with neutrophilic infiltration in orohypopharynx carcinoma tissues**. Consecutive tumor tissue sections from 37 orohypopharynx carcinoma patients were stained against CORTACTIN/CD66b or phospho-CORTACTIN/CD66b. Total CORTACTIN, phospho-CORTACTIN, and neutrophilic infiltration were analyzed with the ImageJ software. **(A)** Correlation between neutrophilic infiltration and the levels of CORTACTIN phosphorylation. Dotted lines indicate the 95% CI (confidence interval) for the regression line. Statistical analysis was performed with Spearman’s rank correlation coefficient (*p* < 0.001; ρ = 0.620). **(B)** The level of CORTACTIN phosphorylation in patients with low (*n* = 19) versus high (*n* = 18) neutrophilic infiltration. Shown are the medians (black lines) and percentiles (25th and 75th) as vertical boxes with error bars; outliers are indicated as small circles. Statistical analysis was performed with the χ^2^ test (*p* = 0.029). **(C)** Representative micrographs (magnification = 200×) of CORTACTIN phosphorylation in patients with low versus high neutrophilic infiltration. Green, CORTACTIN or phospho-CORTACTIN; red, CD66b (neutrophils); blue pseudocolor, DRAQ5 (nuclear staining).

Next, we tested whether neutrophil-induced phosphorylation of CORTACTIN might occur also *in vivo*. To this end, we stained consecutive sections from frozen orohypopharynx carcinoma tissues (37 patients) against total and phosphorylated (Tyr421) forms of CORTACTIN, respectively. All samples were co-stained against CD66b (neutrophil marker). Micrographs were taken at 100-fold magnification from the same region of the tumor tissue for CORTACTIN or phospho-CORTACTIN (each with CD66b co-staining). The micrographs were analyzed with the ImageJ software and the values obtained were considered as Arbitrary Fluorescent Units (AFU). We then plotted the levels of CORTACTIN phosphorylation [phospho-CORTACTIN (AFU)] against the levels of neutrophilic infiltration [CD66b (AFU); Figure 6A]. Statistical analysis using Spearman’s rank test indicated that CORTACTIN phosphorylation significantly correlated with neutrophilic infiltration (*p* < 0.001, ρ = 0.620). Additionally, we divided the patients into CD66b^low^ and CD66b^high^ according to the median value of CD66b (AFU) and found that CD66b^high^ patients had significantly higher CORTACTIN phosphorylation than CD66b^low^ patients (*p* = 0.029, χ^2^; Figure 6B). A representative example of CORTACTIN phosphorylation in CD66b^low^ versus CD66b^high^ patients is shown in Figure 6C. Taken together, our *in vitro* and *in situ* studies demonstrate that neutrophils interact with the tumor cells by inducing CORTACTIN phosphorylation and activation.

### Tumoral CORTACTIN – neutrophil interaction: Functional consequences

**Figure 7:**
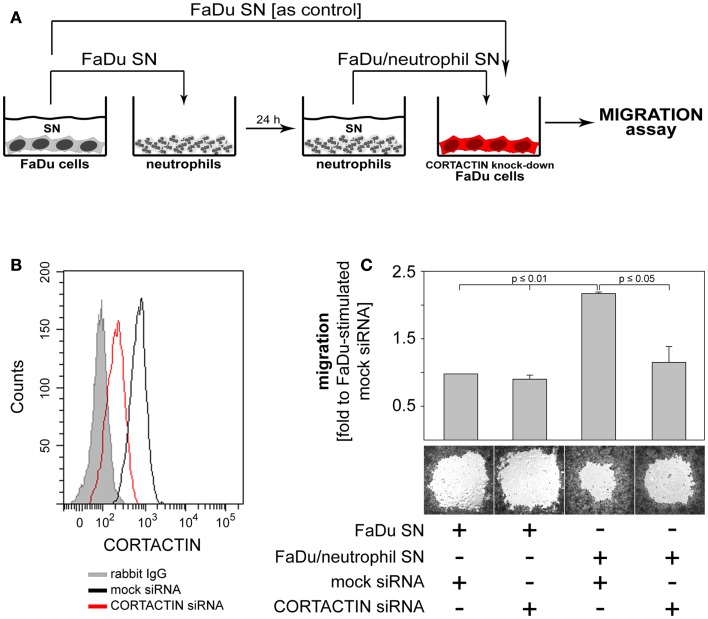
**Neutrophils promote the migration of FaDu cells via CORTACTIN**. **(A)** FaDu cells in which CORTACTIN had been knocked-down by siRNA were stimulated with FaDu/neutrophil SN and migration was assessed 48 h later. **(B)** Efficiency of CORTACTIN knock-down was determined by flow cytometric analysis of total CORTACTIN levels at 72 h post-transfection. **(C)** FaDu cells (transfected with CORTACTIN or mock siRNA) were grown to confluency in the presence of “gap”-forming culture inserts. The “gap” refers to the cell-free round area visible in the figure. Stimulation was performed as indicated in A. Cells were fixed/stained with a solution containing crystal violet and micrographs were taken at 25-fold magnification. “Gap”-closure was quantified with the ImageJ software. Data are means ± SD of three independent experiments (upper panel). A representative result is shown in the lower panel of the figure. Statistical testing was performed with the two-tailed paired Student’s *t*-test.

Finally, we investigated which functional changes might neutrophils induce in the tumor cells via CORTACTIN. Based on the findings that: (i) CORTACTIN is an important regulator of cellular migration (Ammer and Weed, 2008) and (ii) neutrophils enhance the migration of HNC cells (Dumitru et al., 2011), we hypothesized that neutrophils “exploit” high tumoral CORTACTIN levels to promote the migration of the tumor cells. Conversely, in the absence of tumoral CORTACTIN, neutrophils would not be able to stimulate the migration of the tumor cells. We, therefore, knocked-down CORTACTIN in FaDu cells by siRNA. As control, FaDu cells were transfected with mock siRNA (Figures 7A,B). We then determined the migratory potential of the tumor cells in the presence or absence of factors released by “primed” neutrophils. The results showed that mock-transfected FaDu cells displayed accelerated migration in response to neutrophil stimulation, as indicated by enhanced closure of the detection zone (“gap”; Figure 7C). In contrast, this effect of neutrophil stimulation was abolished in CORTACTIN knock-down FaDu cells (Figure 7C). These findings indicate that, consistent with the results presented in Figure 4, high levels of tumoral CORTACTIN are required for neutrophil-induced tumoral migration and progression.

## Discussion

Increased effort has been made to identify cellular/molecular factors that could provide accurate information regarding cancer diagnosis, prognosis, and response to therapy. In this study, we identify tumoral CORTACTIN as a prognostic factor for the clinical outcome in orohypopharynx carcinoma patients. More importantly, we demonstrate that combined analysis of tumoral CORTACTIN and of neutrophilic infiltration in the tumor tissue has higher prognostic impact for the clinical outcome in these patients. In addition to the clinical findings, our study provides a biological and mechanistical link between neutrophils and tumoral CORTACTIN by showing that neutrophils phosphorylate CORTACTIN in the tumor cells to promote their migration.

The potential role of CORTACTIN as prognostic biomarker has been previously investigated in different types of cancer (Li et al., 2008; Wang et al., 2009; Cai et al., 2010; Xu et al., 2010). Several studies observed that CORTACTIN associated with poor clinical outcome also in HNC patients (Gibcus et al., 2008; Hofman et al., 2008; Rodrigo et al., 2009). Among the HNC subtypes, CORTACTIN has been mainly proposed as a potential biomarker for laryngeal carcinoma (Gibcus et al., 2008; Rodrigo et al., 2009). Surprisingly, the study of Rodrigo et al. (2009) suggested that, while having prognostic value in laryngeal carcinoma, CORTACTIN does not associate with the clinical outcome in pharyngeal carcinoma, which is in contrast to our findings. This discrepancy might result from the different read-outs used to assess CORTACTIN (over)expression. While we analyzed directly the protein levels of CORTACTIN in relation to clinical parameters, the above-mentioned studies based their statistical analysis mainly on the gene amplification of CORTACTIN. However, it has been often shown that protein and gene expression do not perfectly correlate. In fact, even the study of Rodrigo et al. (2009) found that gene amplification of CORTACTIN did not correlate to the protein levels observed by immunohistochemistry in 35% of specimens. Yet proteins are the ultimate effectors in biological processes and analysis of protein levels is likely to provide more reliable biological insights than analysis of gene expression. Thus, by quantifying the protein expression of CORTACTIN, our study sheds a new light on the role of this molecule in the prognosis of HNC.

Of novelty and significance are our findings regarding the interaction between tumoral CORTACTIN and neutrophils (CD66b-positive cells) in orohypopharynx carcinoma. As outlined above, an association of either CORTACTIN or tumor-infiltrating neutrophils with poor clinical outcome in cancer patients has already been proposed by previous studies (Donskov and von der Maase, 2006; Jensen et al., 2009, 2012; Kuang et al., 2011; Trellakis et al., 2011; Ilie et al., 2012). However, a link between CORTACTIN and neutrophils has not been shown in any type of cancer thus far. Here, we demonstrate that patients exhibiting high levels of tumoral CORTACTIN together with high neutrophilic infiltration (CORTACTIN^high^/CD66b^high^) have worse clinical outcome than all other groups of patients in our cohort. These findings are further substantiated by multivariate analysis adjusted for factors potentially relevant for progression of orohypopharynx carcinoma, such as smoking/alcohol consumption, type of therapy, and gender. Within this model, CORTACTIN^high^/CD66b^high^ phenotype was the only significant predictor of poor survival for orohypopharynx carcinoma patients. Although necessary to be confirmed by a larger study incorporating all REMARK guidelines, CORTACTIN, and tumor-infiltrating neutrophils seem, nevertheless, to be promising biomarker candidates for an accurate prognosis in orohypopharynx carcinoma.

Our clinical findings also suggest that neutrophils (indirectly) interact with the tumor cells via CORTACTIN to enhance the progression of orohypopharynx carcinoma. This interaction is confirmed by our *in vitro* and *in situ* studies which show that neutrophils activate (phosphorylate) tumoral CORTACTIN to promote the migration of the tumor cells. These findings are highly relevant, since the molecular mechanisms of tumor-neutrophil interactions are still not completely understood. Previous studies showed that neutrophils have pro-angiogenic effects, or promote migration, invasion, and proliferation of the tumor cells (Gregory and Houghton, 2011; Dumitru et al., 2012b). With regard to regulation of tumoral motility/migration by neutrophils, several potential mechanisms have been recently proposed. In breast carcinoma, neutrophils were shown to cluster tumoral ICAM-1 and phosphorylate FAK (focal adhesion kinase) and paxillin via src, as well as p38-MAPK via Rho-GTPase (Strell et al., 2010). In hepatocellular carcinoma, neutrophils were activated by tumor-derived hyaluronan binding to TLR4 on their surface and promoted tumor cell motility (Wu et al., 2011). Notably, both of the above-mentioned studies indicated that the pro-migratory effects of neutrophils are mediated via direct cell-to-cell contact. In our system, neutrophils activated CORTACTIN and enhanced tumor migration via release of soluble factors. Thus, besides supporting the association of CORTACTIN and neutrophils with increased metastasis in cancer patients, these findings unravel a novel mechanism of neutrophil-mediated tumor migration and progression. We certainly do not exclude that, other infiltrating immune cells (in particular macrophages) enhance the aggressiveness of orohypopharynx carcinoma *in vivo*. However, extensive work is still required to dissect the relative importance of various immune cell subsets in progression of solid cancers.

In summary, our study combines clinical and experimental approaches to provide evidence for a clinically relevant mechanism of tumor immunomodulation by stromal neutrophils. Ultimately, these findings contribute to a better understanding of the tumor microenvironment and foster the development of improved therapeutic strategies against orohypopharynx carcinoma and, perhaps, other types of solid cancer as well.

## Conflict of Interest Statement

The authors declare that the research was conducted in the absence of any commercial or financial relationships that could be construed as a potential conflict of interest.
